# Female genital mutilation/cutting (FGM/C) coding capacities in Swiss university hospitals using the International Classification of Diseases (ICD)

**DOI:** 10.1186/s12889-021-11160-6

**Published:** 2021-06-16

**Authors:** S. Cottler-Casanova, M. Horowicz, A. Gayet-Ageron, J. Abdulcadir

**Affiliations:** 1 Division of Gynaecology, Department of the Woman, the Child and the Adolescent, Geneva University Hospitals, 30 Bld de la Cluse, 1211 Geneva, Switzerland; 2 Department of Epidemiology and Public Health, Swiss Tropical and Public Health Institute, Basel, Switzerland; 3 University of Basel, Basel, Switzerland; 4 Faculty of Medicine, University of Geneva, Geneva, Switzerland; 5 CRC & Division of clinical-epidemiology, Department of health and community medicine, University of Geneva & University Hospitals of Geneva, Geneva, Switzerland

**Keywords:** Female genital mutilation, Female genital cutting, Female genital mutilation/cutting, Indirect estimates, Prevalence, Coding, International classification of diseases, ICD, Switzerland

## Abstract

**Background:**

The real prevalence and incidence of women living with or at risk of female genital mutilation/cutting (FGM/C) is unknown in Switzerland and many parts of Europe, as there are no representative surveys similar to DHS or MICS for European countries. Indirect estimates are commonly used to estimate the number of women with FGM/C in high-income countries, but may not reflect the actual FGM/C prevalence among migrants. Direct measures may provide more accurate estimates that could guide policy- and clinical decision-making. Swiss hospital data may provide a sample of patients that can be used to describe the prevalence of FGM/C in Swiss hospitals. Our study assesses the number of inpatient women and girls in Swiss university hospitals from countries with high FGM/C prevalence, and of inpatients with a coded diagnosis of FGM/C.

**Methods:**

We conducted an exploratory descriptive study in Switzerland to assess the number of women and girls admitted to Swiss university hospitals between 2016 and 2018 from 30 FGM/C practicing countries, as well as inpatients with a coded diagnosis of FGM/C using anonymized data. We calculated indirect estimates for inpatient women and girls living with or at risk of FGM/C and compared them with the number of inpatients with a coded diagnosis of FGM/C.

**Results:**

8720 women and girls from FGM/C practicing countries were admitted. 207 patients had a coded diagnosis of FGM/C, including 7 with a nationality outside the 30 targeted countries, corresponding to an overall prevalence of 2.3% (95%CI, 2.0–2.6). The number of FGM/C cases by hospital was significantly different across years (*P* < 0.001), with a higher proportion of cases collected in Geneva, Switzerland.

**Conclusions:**

The comparison between indirect estimates of inpatients with or at risk of FGM/C and the low number of FGM/C cases coded, suggests low recording and coding capacities of FGM/C.

**Tweetable abstract:**

The capacity of coding primary and secondary diagnosis of FGM/C in Swiss university hospitals seems low.

Protocol number: 2018–01851: SwissEthics Committee, Canton of Geneva, Switzerland.

**Supplementary Information:**

The online version contains supplementary material available at 10.1186/s12889-021-11160-6.

## Introduction

Female genital mutilation/cutting (FGM/C) is the partial or total removal of the external female genitalia for non-medical reasons [1]. The World Health Organization describes four FGM/C types [Table 1]. Approximately 200 million women and girls have undergone the practice according to UNICEF [2]. The Demographic Health Survey (DHS) developed by ICF International or the Multiple Indicator Cluster Surveys (MICS) directed by UNICEF, conducted in 27 African and three Asian countries practicing FGM/C provide FGM/C prevalence estimates based on nationally representative data [4]. These estimates do not include women and girls living with FGM/C who emigrated from FGM/C practicing countries [4]. In the European Union (EU), there were an estimated 578,068 women and girls living with FGM/C in 2011 [5], and 21,706 in Switzerland in 2018 [6] based on indirect measures, where the number of migrant women from a FGM/C practicing country is multiplied by the FGM/C prevalence rate from the same country. The European Institute for Gender Equality estimated the number of migrant girls (0–18) from FGM/C practicing countries at risk of FGM/C as 44,106 in France (2014); 18,339 in Italy (2016); 6122 in Belgium (2016), and a few hundred in Greece, Cyprus, Malta, Ireland, Portugal and Sweden [7, 8].

**Table 1:** Classification of FGM/C according to WHO [3]. When WHO refers to “glans of the clitoris”, part of the body of the clitoris can also be affected

**Type I**	Partial or total removal of the clitoral glans (the external and visible part of the clitoris, which is a sensitive part of the female genitals, with the function of providing sexual pleasure to the woman), and/or the prepuce/clitoral hood (the fold of skin surrounding the clitoral glans).
Type Ia	Removal of the prepuce/clitoral hood only
Type Ib	Removal of the clitoral glans with the prepuce/clitoral hood
**Type II**	Partial or total removal of the clitoral glans and the labia minora, with or without removal of the labia majora.
Type IIa	Removal of the labia minora only
Type IIb	Partial or total removal of the glans of the clitoris and the labia minora
Type IIc	Partial or total removal of the glans of the clitoris, the labia minora and the labia majora
**Type III (Infibulation)**	Narrowing of the vaginal opening with the creation of a covering seal. The seal is formed by cutting and repositioning the labia minora, or labia majora. The covering of the vaginal opening is done with or without removal of the clitoral prepuce/clitoral hood and glans.
Type IIIa	Removal and apposition of the labia minora
Type IIIb	Removal and apposition of the labia majora
**Type IV**	All other harmful procedures to the female genitalia for non-medical purposes, for example, pricking, piercing, incising, scraping and cauterization.

Indirect estimation is a systematic and affordable method for estimating the number of women with FGM/C in high-income countries, in the assumption that the prevalence of FGM/C among migrants does not significantly differ from prevalence among non-migrants [9–11]. However, due to several reasons, including cultural change and varying socioeconomic, and ethnic origins of migrants, it may not reflect the actual FGM/C prevalence in migrants’ country of residence or community [12, 13]. The real prevalence and incidence of FGM/C and the number of minors at risk remains unknown in many countries.

Direct measures may provide more accurate estimates that could guide policy- and clinical decision-making. Surveying samples of migrants to estimate FGM/C prevalence also has limitations, as they might not know whether they experienced FGM/C or be unaware of the type [14]. Swiss hospital data may provide a sample of patients that can be used to describe the prevalence of FGM/C in Swiss hospitals. Furthermore, hospital data represents an opportunity to study access and quality of care for patients who underwent FGM/C, providing guidance for health interventions [15–17].

No data are available on the number of women and girls with FGM/C in Swiss hospitals. No accurate information is available on Swiss healthcare professionals’ capacities to record FGM/C and deal with its complications and prevention. Weak capacities in diagnosis, recording and coding represent the major obstacle to studying hospital data on FGM/C. Studies from Switzerland and other high- and low-income countries, among midwives, gynecologists and obstetricians, general and travel medicine practitioners have shown difficulties in screening, diagnosing, classifying and recording FGM/C [18–23]. Pediatricians also lack training on FGM/C and rarely perform external genital examinations [24, 25].

In this manuscript, we aim to:

1. Assess the number of women and girls from FGM/C practicing countries admitted to Swiss university hospitals.
2. Estimate, using indirect measures, the potential number of inpatients who are possibly living with FGM/C.
3. Measure the number of inpatients with a coded primary or secondary diagnosis of FGM/C. The comparison between indirect estimates of inpatients with FGM/C and the number of FGM/C cases coded in the same hospitals, can inform the diagnostic, recording and coding capacities of FGM/C in Swiss university hospitals.

## Methods

This cross-sectional study was part of a larger research study approved in December, 2018: protocol number 2018–01851 by the Swiss Ethics Committees (SwissEthics) and conducted according to the protocol, the Swiss legal requirements, and the World Medical Association Declaration of Helsinki. An exemption of informed consent was granted by the state of Geneva Swiss Ethics committee for the use of anonymized data extracted from the university hospitals databases. We first calculated the indirect estimates of women and girls living with FGM/C in Switzerland between 2010 and 2018 [6]. We used a similar methodology to Yoder and Van Baelen [5, 26], applying the most recent FGM/C DHS and MICS prevalence figures for each year (for girls and women aged 15–49) from FGM/C practicing countries to the number of migrant women and girls living in Switzerland. We applied the total country prevalence estimates of women aged 15–49 to all migrant women and girls living in Switzerland from the same countries. We also conducted a separate analysis for girls aged 0–14, where we applied the prevalence estimates of girls 0–14 to all migrant girls 0–14 living in Switzerland from the same countries. Where no prevalence estimates for girls 0–14 were available, we applied the prevalence estimates for girls 15–19. Full details are available in another paper [6] [Tables S1 & S2].

Secondly, in February 2019, we asked the five Swiss university hospitals to provide anonymized data for all inpatient women and girls with a nationality from the 30 FGM/C practicing countries [Table 2], and for all inpatients with a diagnosis of FGM/C between 2016 and 2018 [Table 3]. Swiss hospital data only provided information on patient’s nationality, and we therefore used this as a proxy for country of origin, discussed in limitations. In Swiss hospitals, healthcare professionals record diagnosis in patients’ electronic medical charts, and professional coders code this information with the tenth edition of the International Classification of Diseases (ICD) [27]. We received data from the University Hospitals of Geneva (HUG), Lausanne (CHUV), Bern (Inselspital) and Zurich (USZ). The University Hospital of Basel did not participate due to logistical difficulties in data provision. The implication is discussed in the conclusion. Analyses were carried out using STATA version 15.

**Table 2:** Total number of women and girls in Swiss university hospitals between 2016 and 2018 from 30 FGM/C practicing countries

Country of origin	ZürichTotal	Lausanne Total	Geneva Total	Bern Total	Total
Benin	2	12	18	5	37
Burkina Faso	3	12	54	21	90
Cameroon	52	256	261	274	843
Central African Republic	1	1	3	3	8
Chad	2	1	2	13	18
Djibouti	0	4	3	0	7
Egypt	20	15	107	101	243
Eritrea	167	295	362	1881	2705
Ethiopia	59	100	123	287	579
Gambia	7	3	8	16	34
Ghana	42	6	53	79	180
Guinea	8	41	101	31	181
Guinea-Bissau	0	14	9	2	25
Indonesia	20	12	33	64	129
Iraq	66	74	164	481	785
Ivory Coast	41	47	146	72	306
Kenya	41	6	63	125	235
Liberia	2	3	5	5	15
Mali	2	1	32	6	41
Mauritania	0	1	27	1	29
Niger	0	4	23	11	38
Nigeria	42	36	88	169	335
Senegal	5	42	195	29	271
Sierra Leone	4	3	20	23	50
Somalia	101	157	233	523	1014
Sudan and South Sudan	30	2	79	63	174
Tanzania	7	1	33	13	54
Togo	3	61	78	36	178
Uganda	3	1	28	24	56
Yemen	12	7	21	20	60
**Grand Total**	**742**	**1218**	**2372**	**4388**	**8720**

**Table 3:** Description of patients with a FGM/C (*n* = 207) as main or secondary diagnosis between 2016 and 2018 in one of four Swiss university hospitals (Geneva, Lausanne, Bern and Zurich)

Variables	2016 (***n*** = 42)	2017 (***n*** = 69)	2018 (***n*** = 96)	***P*** value
Center, n (%)				< 0.001^a^
Geneva	20 (47.6)	24 (34.8)	67 (69.8)	
Lausanne	13 (31.0)	10 (14.5)	19 (19.8)	
Bern	3 (7.1)	23 (33.3)	6 (6.3)	
Zurich	6 (14.3)	12 (17.4)	4 (4.2)	
Country of origin, n (%)				0.097^a^
Benin	0 (0)	0 (0)	1 (1.0)	
Burkina Faso	1 (2.4)	2 (2.9)	0 (0)	
Cameroon	1 (2.4)	0 (0)	0 (0)	
Egypt	0 (0)	0 (0)	5 (5.2)	
Eritrea	12 (28.6)	37 (53.6)	36 (37.5)	
Ethiopia	2 (4.8)	3 (4.4)	2 (2.1)	
Guinea	0 (0)	0 (0)	6 (6.2)	
Guinea-Bissau	0 (0)	0 (0)	2 (2.1)	
Ivory Coast	1 (2.4)	1 (1.5)	1 (1.0)	
Mali	0 (0)	0 (0)	1 (1.0)	
Mauritania	0 (0)	0 (0)	1 (1.0)	
Nigeria	1 (2.4)	1 (1.5)	3 (3.1)	
Senegal	0 (0)	0 (0)	3 (3.1)	
Somalia	14 (33.3)	18 (26.1)	22 (22.9)	
Sudan and South Sudan	1 (2.4)	1 (1.5)	3 (3.1)	
Unknown or other	9 (21.4)	6 (8.7)	10 (10.4)	
Service, n (%)				< 0.001^a^
Gynecology	13 (31.0)	12 (17.4)	9 (9.4)	
Gynecology or Obstetrics*	1 (2.4)	23 (33.3)	6 (6.3)	
Obstetrics	23 (54.8)	33 (47.8)	79 (82.3)	
Others	5 (11.9)	1 (1.5)	2 (2.1)	
Mean age at first visit (±SD, median)	30.7 (±12.0, 27)	27.7 (±6.1, 27.4)	29.8 (±6.7, 30)	0.162^b^
FGM/C type, n (%)				0.116^b^
Type I	3 (7.1)	13 (18.8)	10 (10.4)	
Type II	8 (19.1)	16 (23.2)	33 (34.4)	
Type III	21 (50.0)	33 (47.8)	39 (40.6)	
Type IV	0 (0)	1 (1.5)	2 (2.1)	
Unspecified or other	10 (23.8)	6 (8.7)	12 (12.5)	
FGM/C type, n (%)				
N90.80 (Female genital mutilation, type unspecified)	3 (7.1)	0 (0)	0 (0)	
N90.81 (FGM, Type I)	3 (7.1)	0 (0)	0 (0)	
N90.82 (FGM, Type II)	8 (19.1)	0 (0)	0 (0)	
N90.83 (FGM, Type III)	21 (50.0)	0 (0)	0 (0)	
N90.88 Other specified non-inflammatory diseases of the vulva and perineum	7 (16.7)	0 (0)	0 (0)	
Z91.70 Personal history of female genital mutilation, type unspecified	0 (0)	6 (8.7)	12 (12.5)	
Z91.71 (FGM, Type I)	0 (0)	13 (18.8)	10 (10.4)	
Z91.72 (FGM, Type II)	0 (0)	16 (23.2)	33 (34.4)	
Z91.73 (FGM, Type III)	0 (0)	33 (47.8	39 (40.6)	
Z91.74 (FGM, Type IV)	0 (0)	1 (1.5)	2 (2.1)	

^a^Fischer’s exact test; ^b^Kruskal-Wallis nonparametric test

* Bern’s datasets did not differentiate gynecological from obstetrical units

The participating hospitals provided data on all inpatient women and girls from the 30 targeted countries and all primary and secondary diagnoses of FGM/C coded between January 1, 2016 and December 31, 2018. Therefore, we estimate indirect prevalence of FGM/C in Swiss hospitals as the proportion of the total number of FGM/C cases recorded on the total number of women and girls from the targeted countries in four Swiss university hospitals between 2016 and 2018. Using the country prevalence estimates of FGM/C among women and girls with a nationality from FGM/C practicing countries in 2016, 2017, and 2018, we then multiplied this prevalence estimates to the total number of inpatient women and girls registered with the same nationality in the hospital database during the same period and obtained an indirect estimation of the number of inpatients with FGM/C in our Swiss hospitals [Table 5]. Inpatients with an FGM/C diagnosis that had a nationality from other countries than the ones targeted were not considered in this estimation.

We provided descriptive statistics with mean, ±standard deviation (SD), and median for continuous variables; number and proportions by categories for qualitative variables. We compared all categorical variables by year and FGM/C type by region (West Africa vs. East Africa) using Chi-2 or Fischer’s exact tests. We compared mean ages by year using non-parametric Kruskal-Wallis test. We estimated FGM/C prevalence within the Swiss university hospital population between 2016 and 2018 and their 95% confidence intervals (95%CIs) using the binomial exact method (Clopper-Pearson method).

## Results

8720 women and girls from countries with high FGM/C prevalence were admitted between 2016 and 2018: 4388 in Bern, 2372 in Geneva, 1218 in Lausanne and 742 in Zurich [Table 2]. Most of them came from Eritrea (31.0%), followed by Somalia in Geneva, Zurich and Bern (11.6%), and Cameroon in Lausanne (9.7%).

207 inpatient women and girls had a coded diagnosis of FGM/C [Table 3]. The number of FGM/C cases by center significantly changed over the years (*P* < 0.001) with more cases in Geneva overall, and it was significantly different by department *(P <* 0.001) with most cases coded in obstetrics. Patients with an FGM/C diagnosis mostly originated from Eritrea (*n* = 85) and Somalia (*n* = 54).

The FGM/C type differed significantly depending on the region of origin *(P =* 0.004): types II and III were significantly more frequent among patients from West Africa and from East Africa, respectively [Table S3].

For all years combined, the calculated FGM/C prevalence was 2.29% (95%CI: 1.98–2.62). We excluded seven patients from CHUV who had a coded diagnosis of FGM/C and were registered as Swiss (n = 4), Ecuadorian (*n* = 1), Turkish (n = 1) and French (n = 1). Thus, outside the 30 targeted countries. FGM/C prevalence significantly increased over time in participating centers: 1.24% in 2016, 2.32% in 2017, and 3.32% in 2018 (*P* < 0.001).

FGM/C prevalence in Swiss hospitals was 3.53% among inpatients from countries with the highest FGM/C prevalence (≥81%), and thus higher than among inpatients from countries with lower FGM/C prevalence (*P* < 0.001). [Table S3]. FGM/C prevalence was significantly higher in women from East Africa (*P* < 0.001).

We applied the FGM/C prevalence among inpatients from each at-risk country separately [Table 5] and indirectly estimated the number of inpatients who could have undergone or be at risk of undergoing FGM/C: 1648 in 2016, 1671 in 2017, and 1628 in 2018 (*n* = 4947).

FGM/C prevalence was lower among minors (0.66%), compared to women above 18 years old (2.46%) (*P* < 0.001) [Tables 4 and 5]. FGM/C prevalence also varied by hospital department, with higher prevalence among inpatients in gynecology and obstetrics (*P* < 0.001). It also varied among women and girls from at-risk countries. It was higher in Geneva, similar in Lausanne and Zürich, and lower in Bern (*P* < 0.001). Prevalence was higher in institutions featuring regular educational programmes about FGM/C and/or a clinic or referral physician for FGM/C.

**Table 4:** Prevalence of FGM/C by age, hospital department, center, and educational programme attendance (*n* = 200 FGM/C)

Variables	Number of cases, n	N	Prevalence, % (95%CI)	***P*** value
Category of age, n (%)				0.001^a^
< 18 years	5	757	0.66 (0.21–1.53)	
> =18 years	195	7936	2.46 (2.13–2.82)	
Hospital department, n (%)				< 0.001^a^
Gynecology & obstetrics	195	4163	4.68 (4.06–5.37)	
Surgery	2	1266	0.16 (0.019–0.57)	
Medical department	1	2362	0.042 (0.0011–0.24)	
Emergency	0	573	0 (0–0.64)	
Pediatrics	2	374	0.53 (0.065–1.92)	
Center, n (%)				< 0.001^b^
Geneva	111	2390	4.64 (3.84–5.57)	< 0.001^b^
Lausanne	35	1218	2.87 (2.01–3.97)	
Bern	32	4388	0.73 (0.50–1.03)	
Zürich	22	742	2.96 (1.87–4.45)	
Educational programme organised, n (%)				
Yes (Geneva, Lausanne)	146	3608	4.05 (3.43–4.74)	
No or unknown (Bern, Zürich)	54	5130	1.05 (0.79–1.37)	

^a^Fischer’s exact test, ^b^Chi-2 test

**Table 5:** Estimated prevalence of FGM/C among inpatients by country and year (*n* = 182 FGM/C, as no nationality was recorded for 18 patients with an FGM/C diagnosis)

Variables		2016					2017					2018			
**Country of origin**	**2016** **FGM/C country prevalence (DHS/MICS)**	**Inpatients, N**	**FGM/C codes expected among inpatients, n**^**a**^	**FGM/C codes among inpatients, n**	**FGM/C prevalence calculated among inpatients, % (95%CI)**	**2017 FGM/C country prevalence (DHS/MICS)**	**Inpatients, N**	**FGM/C codes expected among inpatients, n**^**a**^	**FGM/C codes among inpatients, n**	**FGM/C prevalence calculated among inpatients, % (95%CI)**	**2018 FGM/C country prevalence (DHS/MICS)**	**Inpatients, N**	**FGM/C codes expected among inpatients, n**^**a**^	**FGM/C codes among inpatients, n**	**FGM/C prevalence calculated among inpatients, % (95%CI)**
Benin	0.092	10	1	0	0 (0–30.85)	0.092	13	1	0	0 (0–24.71)	0.092	14	1	1	7.14 (0.18–33.87)
Burkina Faso	0.758	35	27	1	2.86 (0.072–14.92)	0.758	24	18	2	8.33 (1.03–26.99)	0.758	31	23	0	0 (0–11.22)
Cameroon	0.014	268	4	1	0.37 (0.009–2.06)	0.014	311	4	0	0 (0–1.18)	0.014	264	4	0	0 (0–1.39)
Central African Republic	0.242	1	0	0	0 (NA)	0.242	3	1	0	0 (0–70.76)	0.242	4	1	0	0 (0–60.24)
Chad	0.384	7	3	0	0 (0–40.96)	0.384	7	3	0	0 (0–40.96)	0.384	4	2	0	0 (0–60.24)
Djibouti	0.931	3	3	0	0 (0–70.76)	0.931	2	2	0	0 (0–84.19)	0.931	2	2	0	0 (0–84.19)
Egypt	0.872	81	71	0	0 (0–4.45)	0.872	79	69	0	0 (0–4.56)	0.872	83	72	5	6.02 (1.98–13.50)
Eritrea	0.83	878	729	12	1.37 (0.71–2.38)	0.83	910	755	37	4.07 (2.88–5.56)	0.83	917	761	36	3.93 (2.76–5.39)
Ethiopia	0.652	210	137	2	0.95 (0.12–3.40)	0.652	188	123	3	1.60 (0.33–4.59)	0.652	181	118	2	1.10 (0.13–3.93)
Gambia	0.749	14	10	0	0 (0–23.16)	0.749	15	11	0	0 (0–21.80)	0.757	5	4	0	0 (0–52.18)
Ghana	0.038	59	2	0	0 (0–6.06)	0.038	69	3	0	0 (0–5.21)	0.038	52	2	0	0 (0–6.85)
Guinea	0.968	43	42	0	0 (0–8.22)	0.968	63	61	0	0 (0–5.69)	0.945	75	71	6	8.00 (2.99–16.60)
Guinea-Bissau	0.449	5	2	0	0 (0–52.18)	0.449	5	2	0	0 (0–52.18)	0.449	15	7	2	13.33 (1.66–40.46)
Iraq	0.367	265	97	0	0 (0–1.38)	0.367	263	97	0	0 (0–1.39)	0.367	257	94	0	0 (0–1.43)
Ivory Coast	0.21	88	18	1	1.14 (0.029–6.17)	0.21	101	21	1	0.99 (0.025–5.39)	0.21	117	25	1	0.85 (0.022–4.67)
Kenya	0.498	81	40	0	0 0–4.45)	0.498	77	38	0	0 (0–4.68)	0.498	77	38	0	0 (0–4.68)
Liberia	0.827	4	3	0	0 (0–60.24)	0.827	4	3	0	0 (0–60.24)	0.886	7	6	0	0 (0–40.96)
Mali	0.666	20	13	0	0 (0–16.84)	0.666	11	7	0	0 (0–28.49)	0.666	10	7	1	10.00 (0.25–44.50)
Mauritania	0.02	11	0	0	0 (0–28.49)	0.02	10	0	0	0 (0–30.85)	0.02	8	0	1	12.50 (0.32–52.65)
Niger	0.184	19	3	0	0 (0–17.65)	0.184	7	1	0	0 (0–40.96)	0.195	12	2	0	0 (0–26.46)
Nigeria	0.227	100	23	1	1.00 (0.025–5.45)	0.24	124	30	1	0.81 (0.020–4.41)	0.24	111	27	3	2.70 (0.56–7.70)
Senegal	0.896	96	86	0	0 (0–3.77)	0.861	91	78	0	0 (0–3.97)	0.861	84	72	3	3.57 (0.74–10.08)
Sierra Leone	0.979	17	17	0	0 (0–19.51)	0.979	19	19	0	0 (0–17.65)	0.979	14	14	0	0 (0–23.16)
Somalia	0.866	352	305	14	3.98 (2.19–6.58)	0.866	358	310	18	5.03 (3.01–7.83)	0.866	304	263	22	7.24 (4.59–10.75)
Sudan and South Sudan	0.1	58	6	1	1.72 (0.044–9.24)	0.1	65	7	1	1.54 (0.039–8.28)	0.1	51	5	3	5.88 (1.23–16.24)
Tanzania	0.047	19	1	0	0 (0–17.65)	0.047	18	1	0	0 (0–18.53)	0.047	17	1	0	0 (0–19.51)
Togo	0.003	69	0	0	0 (0–5.21)	0.003	53	0	0	0 (0–84.19)	0.003	56	0	0	0 (0–6.38)
Uganda	0.185	15	3	0	0 (0–21.80)	0.185	21	4	0	0 (0–16.11)	0.185	20	4	0	0 (0–16.11)
Yemen	0.092	24	2	0	0 (0–14.25)	0.092	15	1	0	0 (0–21.80)	0.092	21	2	0	0 (0–16.11)
**Total**	**NA**	**2892**	**1648**	**33**	**NA**	**NA**	**2973**	**1671**	**63**	**NA**	**NA**	**2855**	**1628**	86	NA

n^a^= rounded to the unit

## Discussion

### Main findings

Our findings show that only 207 patients (2.29%) have a coded FGM/C diagnosis, with an increase between 2016 and 2018. There is a drastic difference between FGM/C cases coded in Swiss university hospitals (n = 207) and the possible number of women and girls with FGM/C in these hospitals based on our indirect estimates (*n* =4947). Our results suggest that FGM/C is not accurately diagnosed, recorded and/or coded in Swiss university hospitals. Moreover, most women and girls came from Eritrea and Somalia, where FGM/C prevalence exceeds 80%, and where type III is frequent, the latter type being easier to identify, and associated with more long-term complications [1]. Infibulation was indeed the most frequent type among inpatients from East Africa.

Seven inpatients with an FGM/C code did not have the nationality of a country where the practice is usually performed. The nationality recorded or FGM/C coding might be incorrect. Alternatively, these women come from FGM/C practicing countries but possess another nationality, and underwent FGM/C before migrating, or afterwards while visiting their country of origin. In such case, and if coding is accurate, monitoring FGM/C prevalence with ICD codes might give more reliable results than indirect estimates and overcome the issue of nationality and ethnicity.

Coding was significantly higher in gynecology and obstetrics compared to other departments. Obstetricians and gynecologists routinely examine the external genitalia and might be more trained to recognize FGM/C. Furthermore, our results suggest that pregnancy and delivery are critical times for diagnosing FGM/C, because it was significantly more coded in obstetrics than in gynecology. Only two girls in pediatrics and two women in urology were coded with FGM/C.

The prevalence of FGM/C codes in minors (0.66%) was significantly lower than in adult women (2.46%). Belonging to a new generation of immigrants, length of stay, and migration in a country where FGM/C is illegal could explain why it is less frequent among minors [7, 8, 12]. However, insufficient screening and routine genital examinations among pediatricians, or absent documentation can also explain the low numbers [23]. A specific code for “risk of FGM/C” might facilitate screening and prevention [17].

**Fig. 1:**
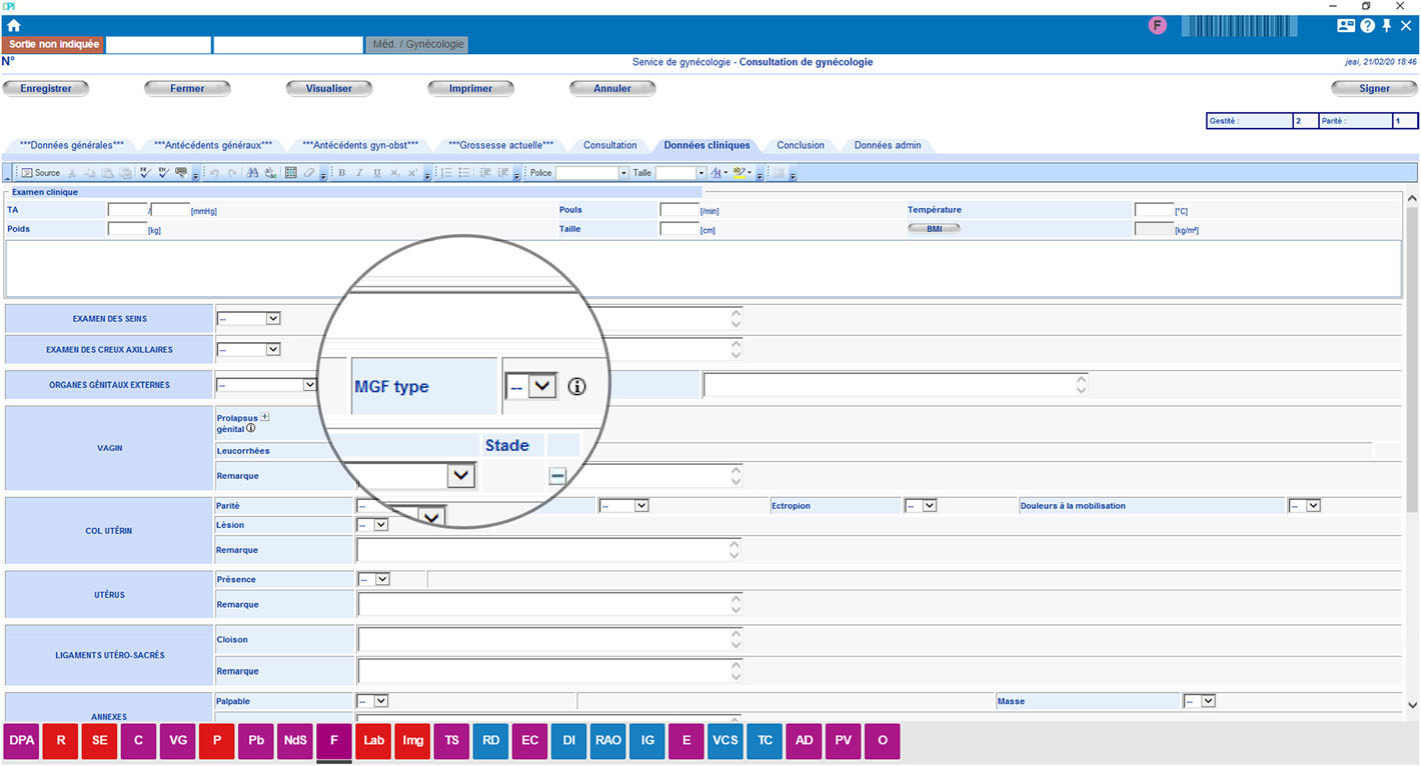
FGM/C checkbox in the gynecological electronic medical chart

**Fig. 2:**
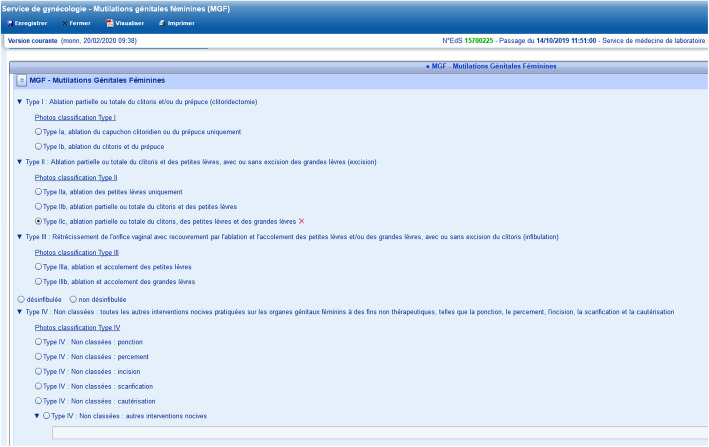
FGM/C type and description in the gynecological electronic medical chart

Longstanding training and protocols about FGM/C in Geneva and Lausanne could explain why FGM/C prevalence in these hospitals was higher than in Bern, even though Bern numbered more patients from FGM/C practicing countries. At HUG for instance, a retrospective review of the medical files of patients who attended the FGM/C outpatient clinic between 2010 and 2012 revealed missed and misclassification of FGM/C in more than one-third of cases [18]. Therefore, the obstetric and gynecologic divisions implemented several interventions: updating the protocols for the care of women and girls with FGM/C, learning tools with drawings, pictures and videos [28], workshops for midwives, and simulation programmes on defibulation. Since 2010, workshops were also organized in pediatrics, travel medicine, HIV clinic, infectious disease, and primary care. In 2017, the HUG hosted an International expert symposium on the care of women and girls with FGM/C and on prevention [29]. In 2012, the HUG’s division of gynecology introduced an FGM/C checkbox in electronic medical forms to record FGM/C and its type. An update in February 2018 [Figs. 1 & 2] added the description of FGM/C types and subtypes in gynecology and obstetrics. This may have facilitated screening and recording, explaining why FGM/C codes almost tripled between 2017 and 2018.

According to a survey run by the Swiss Network against Female Circumcision in 2017 (unpublished, data obtained from the authors), FGM/C was taught at the medical faculties of the Universities of Geneva, Lausanne and Fribourg but not in Bern, whose university hospital admitted 4388 women and girls from FGM/C practicing countries between 2016 and 2018. This might partially explain the higher FGM/C prevalence in Geneva and Lausanne. Zürich’s medical faculty did not reply to this survey.

### Strengths and limitations

One strength of the study was the use of ICD-10 codes for estimating FGM/C prevalence, making it easily reproducible to allow comparison of data over time, and after specific interventions [17].

The main weakness is that we could only collect data of patients, for whom FGM/C had been coded, and undercoding is evident. FGM/C is probably not recorded/coded when the reason for hospitalization and FGM/C are not related. However, even when they are related, FGM/C recording/coding is probably missing: the calculated prevalence of FGM/C among 4163 inpatient women in obstetrics and gynecology is only 4.68%.

We included inpatients registered with the nationality from one of 30 FGM/C practicing countries, irrespective of whether they were first- or second- (or third-) generation migrants. This may exclude women who might originally come from one of these countries but have now a different nationality.

We did not have the information regarding the age of all inpatient women in the anonymized data. However, the aim of our study was not to assess the prevalence by 5-year age groupings as is often done in high FGM/C prevalence countries to assess the evolution of the practice. Even though FGM/C is age-dependent, it is generally performed before menarche [30] and before migrating [31, 32]. Furthermore, in Swiss university hospitals, inpatients that are less than 16 years old are generally hospitalized in the pediatric division. We can hypothesize that all inpatients except for those from pediatric departments are more than 16 years old, and are therefore likely over the age of cutting. Future research reproducing our methodology might analyze the age of the women and girls included. We can hypothesize that a considerable number of inpatient women in Swiss university hospitals are mainly women of childbearing age as they were mostly attended in gynecology or obstetrics units.

We limited our study to four Swiss university hospitals. Basel’s University Hospital could not provide the data requested, but we hypothesize that we would have found equally low FGM/C prevalence. We did not study regions without university hospitals, such as Tessin, on the Italian border, where hospitals could admit migrant women and girls with or at risk of FGM/C [9].

We included hospitalized patients only. It would be interesting to analyze data of outpatient women and girls in pediatrics, travel medicine, infectious disease, primary care services, and migrants’ physical and mental health programmes.

### Interpretation

The number of inpatients with an FGM/C diagnosis out of all women and girls potentially living with FGM/C is low in all hospitals and specialties, including gynecology and obstetrics. We believe that FGM/C coding indirectly reflects awareness of the phenomenon. If FGM/C is not recognized or discussed, women and girls living with FGM/C cannot access specific care, health and legal information and prevention.

Our study suggests that training healthcare professionals and medical students increases the number of patients coded with FGM/C. A study conducted in Belgium showed that more patients were coded with FGM/C after delivering information on FGM/C and its management [33]. The introduction of an FGM/C checkbox in electronic medical charts also seems to facilitate the diagnosis. Similar use of electronic tools facilitated identification of intimate partner violence, together with routine protocols on appropriate screening and counseling [34]. Since November 2019, at HUG, the FGM/C checkbox is linked to a standardized form where physicians, nurses and midwives can record the type, subtype and complications identified, and access an illustrated description from a learning tool for each item [28].

## Conclusion

The present study shows that assessing FGM/C coding through ICD-10 is feasible but FGM/C coding capacities among inpatients in Swiss university hospitals are low.

Future policies should include training on appropriate screening, diagnosis, management and referral in case of FGM/C. Training should be organized in different specialties such as urology, obstetrics and gynecology, infectious diseases, general practice, pediatrics and psychiatry [35], and stress the importance of recording and coding. Certified interpreters and coders should also receive training. Finally, professionals in obstetrics, pediatrics, primary care, and travel medicine should be able to identify children at risk and discuss prevention, national laws on FGM/C and child’s rights [22–25, 36]. Sensitizing and teaching about FGM/C in existing pre-graduate classes, such as anatomy, gynecology and obstetrics, urology, infectious disease, pediatrics, psychiatry and primary care could improve standard training.

Our next step is to assess knowledge, attitudes and practice of healthcare professionals in the same hospitals to tailor training programmes and tools that can improve screening, prevention, diagnosis and management of FGM/C. We will also analyze our data according to the belonging of the included institutions to *Swiss Hospitals for equity*, a network aiming at improving healthcare access for underprivileged groups, regardless of their origin, language and socioeconomic situation. Routine availability of certified interpreters, like in Geneva and Lausanne, might facilitate diagnosis, recording and coding of FGM/C.

## Supplementary Information

**Additional file 1.**

## Data Availability

The datasets used and/or analysed during the current study available from the corresponding author on reasonable request.
