# Supplementary material for: Female genital mutilation/cutting (FGM/C) coding capacities in Swiss university hospitals using the International Classification of Diseases (ICD)
Source: BMC Public Health. 2021 Jun 16;21:1151. doi: 10.1186/s12889-021-11160-6 (PMC8207741; doi:10.1186/s12889-021-11160-6)
Supplement: Supplementary file 1 — Additional file 1. [file 12889_2021_11160_MOESM1_ESM.docx]

**Female Genital Mutilation/Cutting (FGM/C) coding capacities in Swiss University Hospitals using the International Classification of Diseases (ICD)**

S. Cottler-Casanova^1,2,3*^, M. Horowicz^4*^, A. Gayet-Ageron^5^, J. Abdulcadir^1CA^

^*^First co-authorship

^1^Division of Gynaecology. Department of the Woman, the Child and the Adolescent.

Geneva University Hospitals

^2^Department of Epidemiology and Public Health, Swiss Tropical and Public Health Institute, Basel, Switzerland;

^3^University of Basel, Basel, Switzerland;

^4^Faculty of Medicine. University of Geneva

^5^CRC & Division of clinical-epidemiology, Department of health and community medicine, University of Geneva & University Hospitals of Geneva

^CA^ Corresponding author: Jasmine Abdulcadir

Division of Gynaecology.

Department of the Woman, the Child and the Adolescent.

Geneva University Hospitals

30 Bld de la Cluse

1211 Geneva, Switzerland

Office 0041-22-3724049

jasmine.abdulcadir@hcuge.ch

Running title: FGM/C coding capacities in Swiss university hospitals

**Table S1.** Indirect prevalence estimates of women and girls living with FGM/C in Switzerland between 2010 and 2018^25^

| Country | Swiss 2010 Applied Indirect Prevalence Estimate [WOMEN & GIRLS] | Swiss 2011 Applied Indirect Prevalence Estimate [WOMEN & GIRLS] | Swiss 2012 Applied Indirect Prevalence Estimate [WOMEN & GIRLS] | Swiss 2013 Applied Indirect Prevalence Estimate [WOMEN & GIRLS] | Swiss 2014 Applied Indirect Prevalence Estimate [WOMEN & GIRLS] | Swiss 2015 Applied Indirect Prevalence Estimate [WOMEN & GIRLS] | Swiss 2016 Applied Indirect Prevalence Estimate [WOMEN & GIRLS] | Swiss 2017 Applied Indirect Prevalence Estimate [WOMEN & GIRLS] | Swiss 2018 Applied Indirect Prevalence Estimate [WOMEN & GIRLS] |
| --- | --- | --- | --- | --- | --- | --- | --- | --- | --- |
| Benin | 14.964 | 9.052 | 10.731 | 11.388 | 13.340 | 13.800 | 13.432 | 13.156 | 11.684 |
| Burkina Faso | 127.344 | 136.440 | 137.198 | 136.440 | 141.746 | 143.262 | 143.262 | 150.842 | 145.536 |
| Cameroon | 37.716 | 38.136 | 38.094 | 38.192 | 38.136 | 38.374 | 38.640 | 38.318 | 37.870 |
| Central African Republic | 6.776 | 6.534 | 7.502 | 5.808 | 6.050 | 5.808 | 7.018 | 7.986 | 7.986 |
| Chad | 26.078 | 25.194 | 27.404 | 32.266 | 27.264 | 26.880 | 26.112 | 24.576 | 24.576 |
| Djibouti | 10.241 | 12.103 | 13.034 | 13.034 | 13.965 | 16.758 | 17.689 | 14.896 | 14.896 |
| Egypt | 608.548 | 670.496 | 693.271 | 740.643 | 762.398 | 721.144 | 727.248 | 754.280 | 753.408 |
| Eritrea | 2953.140 | 4164.110 | 6076.430 | 6962.040 | 8549.000 | 10672.970 | 11901.370 | 12948.000 | 13730.690 |
| Ethiopia | 1110.785 | 1140.505 | 1239.324 | 1316.596 | 1372.321 | 1445.878 | 1336.600 | 1353.552 | 1365.940 |
| Gambia | 48.832 | 51.884 | 65.618 | 73.402 | 79.394 | 92.127 | 90.629 | 97.370 | 106.737 |
| Ghana | 26.334 | 26.144 | 26.638 | 26.866 | 27.170 | 26.904 | 27.132 | 27.170 | 27.474 |
| Guinea | 205.540 | 218.924 | 251.940 | 260.661 | 278.103 | 289.731 | 308.792 | 324.280 | 342.090 |
| Guinea-Bissau | 9.462 | 16.932 | 22.410 | 24.402 | 21.103 | 24.246 | 23.797 | 24.246 | 27.389 |
| Iraq | 224.210 | 228.501 | 226.314 | 227.529 | 234.981 | 282.690 | 301.239 | 301.725 | 281.496 |
| Ivory Coast | 322.140 | 351.822 | 365.956 | 372.832 | 375.888 | 373.596 | 356.357 | 369.202 | 369.936 |
| Kenya | 234.957 | 245.797 | 249.862 | 253.114 | 204.120 | 209.790 | 214.830 | 215.880 | 225.120 |
| Liberia | 37.248 | 38.412 | 40.158 | 34.362 | 31.374 | 30.876 | 29.880 | 29.382 | 27.888 |
| Mali | 93.810 | 91.155 | 101.775 | 106.938 | 110.594 | 86.008 | 89.316 | 89.316 | 101.890 |
| Mauritania | 21.660 | 24.290 | 24.290 | 21.514 | 21.514 | 18.648 | 17.982 | 21.978 | 8.986 |
| Niger | 0.902 | 0.858 | 0.800 | 0.840 | 0.880 | 0.960 | 0.840 | 0.820 | 0.980 |
| Nigeria | 205.720 | 214.650 | 237.600 | 212.536 | 220.472 | 223.200 | 167.256 | 177.928 | 192.465 |
| Senegal | 127.986 | 134.154 | 140.579 | 145.719 | 145.977 | 152.944 | 148.912 | 162.000 | 161.280 |
| Sierra Leone | 69.520 | 62.480 | 66.000 | 60.928 | 57.344 | 68.096 | 63.616 | 63.714 | 61.992 |
| Somalia | 2218.414 | 2345.684 | 2592.392 | 2648.195 | 2759.801 | 2967.349 | 3174.897 | 3206.225 | 3220.910 |
| Sudanb | 224.256 | 240.024 | 253.164 | 250.536 | 270.192 | 280.584 | 323.018 | 323.018 | 354.194 |
| Tanzania | 23.798 | 24.090 | 23.652 | 24.820 | 25.404 | 17.700 | 18.400 | 18.300 | 18.800 |
| Togo | 22.152 | 23.361 | 24.492 | 30.503 | 31.960 | 32.759 | 32.853 | 32.336 | 32.289 |
| Uganda | 1.332 | 2.996 | 3.066 | 3.108 | 2.996 | 2.856 | 0.630 | 0.705 | 0.759 |
| Yemen | 45.150 | 48.375 | 51.385 | 46.250 | 50.135 | 51.245 | 49.210 | 49.950 | 51.430 |
| Total | 9059.01 | 10593.10 | 13011.079 | 14081.462 | 15873.622 | 18317.183 | 19650.957 | 20841.151 | 21706.691 |

**Table S2.** Type of FGM/C and region (n=182 FGM/C, as no nationality was recorded for 18 patients with an

FGM/C diagnosis).

| **Variables** | **West Africa**  **(n=26)** | **East Africa**  **(n=156)** | ***P* value^a^** |
| --- | --- | --- | --- |
| FGM/C type, n (%)  Type I  Type II  Type III  Type IV  Unspecified or other | 5 (19.2)  14 (53.9)  3 (11.5)  0 (0)  4 (15.4) | 17 (10.9)  41 (26.3)  79 (50.6)  3 (1.9)  16 (10.3) | 0.004 |

^a^Fischer’s exact test

**Table S3.** Prevalence of FGM/C by region and categories of country prevalence (n=182 FGM/C, as no nationality

was recorded for 18 patients with an FGM/C diagnosis).

| **Variables** | **Number of cases, n** | **N** | **Prevalence, % (95%CI)** | ***P* value^a^** |
| --- | --- | --- | --- | --- |
| Region, n (%)  West Africa  Central Africa  East Africa  Middle East  Asia | 26  0  156  0  0 | 2653  26  5067  845  129 | 0.98 (0.64-1.43)  0 (0-13.23)  3.08 (2.62-3.59)  0 (0-0.44)  0 (0-2.82) | <0.001 |
| Country prevalence, n (%)  >=81%  51-80%  26-50%  10-25%  <10% | 156  11  13  1  1 | 4415  876  1190  159  2080 | 3.53 (3.01-4.12)  1.26 (0.63-2.24)  1.09 (0.58-1.86)  0.63 (0.016-3.45)  0.048 (0.0012-0.27) | <0.001 |
| Country of origin, n (%)  Benin  Burkina Faso  Cameroon  Egypt  Eritrea  Ethiopia  Guinea  Guinea-Bissau  Ivory Coast  Mali  Mauritania  Nigeria  Senegal  Somalia  Sudan and South Sudan  Other | 1  3  1  5  85  7  6  2  3  1  1  5  3  54  5  0 | 37  90  843  243  2705  579  181  25  306  41  29  335  271  1014  174  1847 | 2.70 (0.068-14.16)  3.33 (0.69-9.43)  0.12 (0.003-0.66)  2.06 (0.67-4.74)  3.14 (2.52-3.87)  1.21 (0.49-2.48)  3.31 (1.23-7.08)  8.00 (0.98-26.03)  0.98 (0.20-2.84)  2.44 (0.061-12.86)  3.45 (0.088-17.76)  1.49 (0.49-3.45)  1.11 (0.23-3.20)  5.33 (4.03-6.89)  2.87 (0.94-6.58)  0 (0-0.20) | <0.001 |

^a^Fischer’s exact test

**Table S4.** Estimated prevalence of FGM/C among inpatients by country and year (n=182 FGM/C, as no nationality was recorded for 18 patients with an FGM/C diagnosis).

|  |  |  | | | |  |  | | | |  |  | | | |
| --- | --- | --- | --- | --- | --- | --- | --- | --- | --- | --- | --- | --- | --- | --- | --- |
| **Variables** |  |  |  |  |  |  |  |  |  |  |  |  |  |  |  |
|  |  |  |  |  |  |  |  |  |  |  |  |  |  |  |  |
|  |  |  |  |  |  |  |  |  |  |  |  |  |  |  |  |
|  |  |  |  |  |  |  |  |  |  |  |  |  |  |  |  |
|  |  |  |  |  |  |  |  |  |  |  |  |  |  |  |  |
|  |  | **2016** | | | |  | **2017** | | | |  | **2018** | | | |
|  |  |  | | | |  |  | | | |  |  | | | |
| **Country of origin** | **2016** | **Inpatients, N** | **FGM/C codes expected among inpatients, n^a^** | **FGM/C codes among inpatients, n** | **FGM/C prevalence calculated among inpatients, % (95%CI)** | **2017 FGM/C country prevalence (DHS/MICS)** | **Inpatients, N** | **FGM/C codes expected among inpatients, n^a^** | **FGM/C codes among inpatients, n** | **FGM/C prevalence calculated among inpatients, % (95%CI)** | **2018 FGM/C country prevalence (DHS/MICS)** | **Inpatients, N** | **FGM/C codes expected among inpatients, n^a^** | **FGM/C codes among inpatients, n** | **FGM/C prevalence calculated among inpatients, % (95%CI)** |
|  | **FGM/C country prevalence (DHS/MICS)** |  |  |  |  |  |  |  |  |  |  |  |  |  |  |
| Benin | 0.092 | 10 | 1 | 0 | 0 (0-30.85) | 0.092 | 13 | 1 | 0 | 0 (0-24.71) | 0.092 | 14 | 1 | 1 | 7.14 (0.18-33.87) |
| Burkina Faso | 0.758 | 35 | 27 | 1 | 2.86 (0.072-14.92) | 0.758 | 24 | 18 | 2 | 8.33 (1.03-26.99) | 0.758 | 31 | 23 | 0 | 0 (0-11.22) |
| Cameroon | 0.014 | 268 | 4 | 1 | 0.37 (0.009-2.06) | 0.014 | 311 | 4 | 0 | 0 (0-1.18) | 0.014 | 264 | 4 | 0 | 0 (0-1.39) |
| Central African Republic | 0.242 | 1 | 0 | 0 | 0 (NA) | 0.242 | 3 | 1 | 0 | 0 (0-70.76) | 0.242 | 4 | 1 | 0 | 0 (0-60.24) |
| Chad | 0.384 | 7 | 3 | 0 | 0 (0-40.96) | 0.384 | 7 | 3 | 0 | 0 (0-40.96) | 0.384 | 4 | 2 | 0 | 0 (0-60.24) |
| Djibouti | 0.931 | 3 | 3 | 0 | 0 (0-70.76) | 0.931 | 2 | 2 | 0 | 0 (0-84.19) | 0.931 | 2 | 2 | 0 | 0 (0-84.19) |
| Egypt | 0.872 | 81 | 71 | 0 | 0 (0-4.45) | 0.872 | 79 | 69 | 0 | 0 (0-4.56) | 0.872 | 83 | 72 | 5 | 6.02 (1.98-13.50) |
| Eritrea | 0.83 | 878 | 729 | 12 | 1.37 (0.71-2.38) | 0.83 | 910 | 755 | 37 | 4.07 (2.88-5.56) | 0.83 | 917 | 761 | 36 | 3.93 (2.76-5.39) |
| Ethiopia | 0.652 | 210 | 137 | 2 | 0.95 (0.12-3.40) | 0.652 | 188 | 123 | 3 | 1.60 (0.33-4.59) | 0.652 | 181 | 118 | 2 | 1.10 (0.13-3.93) |
| Gambia | 0.749 | 14 | 10 | 0 | 0 (0-23.16) | 0.749 | 15 | 11 | 0 | 0 (0-21.80) | 0.757 | 5 | 4 | 0 | 0 (0-52.18) |
| Ghana | 0.038 | 59 | 2 | 0 | 0 (0-6.06) | 0.038 | 69 | 3 | 0 | 0 (0-5.21) | 0.038 | 52 | 2 | 0 | 0 (0-6.85) |
| Guinea | 0.968 | 43 | 42 | 0 | 0 (0-8.22) | 0.968 | 63 | 61 | 0 | 0 (0-5.69) | 0.945 | 75 | 71 | 6 | 8.00 (2.99-16.60) |
| Guinea-Bissau | 0.449 | 5 | 2 | 0 | 0 (0-52.18) | 0.449 | 5 | 2 | 0 | 0 (0-52.18) | 0.449 | 15 | 7 | 2 | 13.33 (1.66-40.46) |
| Iraq | 0.367 | 265 | 97 | 0 | 0 (0-1.38) | 0.367 | 263 | 97 | 0 | 0 (0-1.39) | 0.367 | 257 | 94 | 0 | 0 (0-1.43) |
| Ivory Coast | 0.21 | 88 | 18 | 1 | 1.14 (0.029-6.17) | 0.21 | 101 | 21 | 1 | 0.99 (0.025-5.39) | 0.21 | 117 | 25 | 1 | 0.85 (0.022-4.67) |
| Kenya | 0.498 | 81 | 40 | 0 | 0 0-4.45) | 0.498 | 77 | 38 | 0 | 0 (0-4.68) | 0.498 | 77 | 38 | 0 | 0 (0-4.68) |
| Liberia | 0.827 | 4 | 3 | 0 | 0 (0-60.24) | 0.827 | 4 | 3 | 0 | 0 (0-60.24) | 0.886 | 7 | 6 | 0 | 0 (0-40.96) |
| Mali | 0.666 | 20 | 13 | 0 | 0 (0-16.84) | 0.666 | 11 | 7 | 0 | 0 (0-28.49) | 0.666 | 10 | 7 | 1 | 10.00 (0.25-44.50) |
| Mauritania | 0.02 | 11 | 0 | 0 | 0 (0-28.49) | 0.02 | 10 | 0 | 0 | 0 (0-30.85) | 0.02 | 8 | 0 | 1 | 12.50 (0.32-52.65) |
| Niger | 0.184 | 19 | 3 | 0 | 0 (0-17.65) | 0.184 | 7 | 1 | 0 | 0 (0-40.96) | 0.195 | 12 | 2 | 0 | 0 (0-26.46) |
| Nigeria | 0.227 | 100 | 23 | 1 | 1.00 (0.025-5.45) | 0.24 | 124 | 30 | 1 | 0.81 (0.020-4.41) | 0.24 | 111 | 27 | 3 | 2.70 (0.56-7.70) |
| Senegal | 0.896 | 96 | 86 | 0 | 0 (0-3.77) | 0.861 | 91 | 78 | 0 | 0 (0-3.97) | 0.861 | 84 | 72 | 3 | 3.57 (0.74-10.08) |
| Sierra Leone | 0.979 | 17 | 17 | 0 | 0 (0-19.51) | 0.979 | 19 | 19 | 0 | 0 (0-17.65) | 0.979 | 14 | 14 | 0 | 0 (0-23.16) |
| Somalia | 0.866 | 352 | 305 | 14 | 3.98 (2.19-6.58) | 0.866 | 358 | 310 | 18 | 5.03 (3.01-7.83) | 0.866 | 304 | 263 | 22 | 7.24 (4.59-10.75) |
| Sudan and South Sudan | 0.1 | 58 | 6 | 1 | 1.72 (0.044-9.24) | 0.1 | 65 | 7 | 1 | 1.54 (0.039-8.28) | 0.1 | 51 | 5 | 3 | 5.88 (1.23-16.24) |
| Tanzania | 0.047 | 19 | 1 | 0 | 0 (0-17.65) | 0.047 | 18 | 1 | 0 | 0 (0-18.53) | 0.047 | 17 | 1 | 0 | 0 (0-19.51) |
| Togo | 0.003 | 69 | 0 | 0 | 0 (0-5.21) | 0.003 | 53 | 0 | 0 | 0 (0-84.19) | 0.003 | 56 | 0 | 0 | 0 (0-6.38) |
| Uganda | 0.185 | 15 | 3 | 0 | 0 (0-21.80) | 0.185 | 21 | 4 | 0 | 0 (0-16.11) | 0.185 | 20 | 4 | 0 | 0 (0-16.11) |
| Yemen | 0.092 | 24 | 2 | 0 | 0 (0-14.25) | 0.092 | 15 | 1 | 0 | 0 (0-21.80) | 0.092 | 21 | 2 | 0 | 0 (0-16.11) |
| **Total** | **NA** | **2892** | **1648.305** | **33** | **NA** | **NA** | **2973** | **1670.577** | **63** | **NA** | **NA** | **2855** | **1627.947** | 86 | NA |

^a^rounded to the unit
